# ELK3 expressed in lymphatic endothelial cells promotes breast cancer progression and metastasis through exosomal miRNAs

**DOI:** 10.1038/s41598-019-44828-6

**Published:** 2019-06-10

**Authors:** Kwang-Soo Kim, Ji-In Park, Nuri Oh, Hyeon-Ju Cho, Ji-Hoon Park, Kyung-Soon Park

**Affiliations:** Department of Biomedical Science, College of Life Science, CHA University, Seongnam-si, Republic of Korea

**Keywords:** Breast cancer, Non-coding RNAs, Cancer microenvironment

## Abstract

Tumor-associated lymphatic vessels (LV) serve as a route of cancer dissemination through the prometastatic crosstalk between lymphatic endothelial cells (LECs) lining the LVs and cancer cells. Compared to blood endothelial cell-derived angiocrine factors, however, LEC-secreted factors in the tumor microenvironment and their roles in tumor metastasis are poorly understood. Here, we report that ELK3 expressed in LECs contributes to the dissemination of cancer cells during tumor growth by providing oncogenic miRNAs to tumor cells through exosomes. We found that conditioned medium from ELK3-suppressed LECs (LCM) lost its ability to promote the migration and invasion of breast cancer cells such as MDA-MB-231, Hs578T and BT20 *in vitro*. Suppression of ELK3 in LECs diminished the ability of LECs to promote tumor growth and metastasis of MDA-MB-231 *in vivo*. Exosomes derived from LECs significantly increased the migration and invasion of MDA-MB-231 *in vitro*, but ELK3 suppression significantly diminished the pro-oncogenic activity of exosomes from LECs. Based on the miRNA expression profiles of LECs and functional analysis, we identified miR-503-3p, miR-4269 and miR-30e-3p as downstream targets of ELK3 in LECs, which cause the above phenotype of cancer cells. These findings strongly suggest that ELK3 expressed in LECs is a major regulator that controls the communication between the tumor microenvironment and tumors to support cancer metastasis.

## Introduction

The “seed and soil hypothesis” proposes that cancer cells function as “seeds” to manipulate the tumor microenvironment, which functions as the “soil”. Therefore, the communication between tumors and organ microenvironments has been recognized as a core means of tumor growth and metastasis. The lymphatic vessels (LV) within the tumor microenvironment is leaky compared to blood vessels; thus, it is considered a primary route of cancer dissemination^1^. Tumors drive lymphatic hyperplasia or lymphangiogenesis through the production of VEGF-C^2^. There is emerging evidence that lymphatic endothelial cells (LECs) in the tumor microenvironment and the LV that is formed by those cells play an important role in tumor growth and metastasis by manipulating the immune response of tumor cells within the tumor microenvironment^3–5^. The dissemination of cancer cells through the LV is caused by the crosstalk between LECs and cancer cells^6^. This crosstalk is also achieved by numerous factors secreted from LECs and their corresponding receptor signaling pathways in cancer cells^7^. Thus, an understanding of the communication between LEC-secreted proteins and the corresponding signaling pathways in the tumor would contribute to the development of a strategy to defeat cancer metastasis^8^. Compared to blood endothelial cell-derived angiocrine factors, however, LEC-secreted factors in the tumor microenvironment and their roles in tumor metastasis are poorly understood.

Exosomes are extracellular organelles that are released into the microenvironment to perform pleiotropic biological functions, including tumor development and metastasis^9^. The size of exosomes is between 30 nm and 150 nm in diameter, and they contain a wide range of functional proteins, mRNAs and miRNAs^10^. The “seed” cancer cells may retain long-term dormancy until the “soil” microenvironment becomes suitable for tumor growth, which is closely related to the function of exosomes in communicating between the “seed” and “soil”. The interaction between the tumor microenvironment and cancer cells that is mediated by exosomal miRNA in the exosome can affect tumor progression^11^.

ELK3 is an ETS domain-containing protein that is capable of forming a ternary complex with DNA and serum response factor (SRF). ELK3 is a transcriptional repressor that switches to a transcriptional activator following phosphorylation by extracellular signal-regulated kinase 1/2 (ERK1/2) in response to Ras signaling^12^. There are several reports about the role of ELK3 in cancer biology. ELK3 is highly expressed in various cancers, including basal-like malignant breast cancers, and it orchestrates metastasis during tumor progression^13–15^. The suppression of ELK3 in MDA-MB-231 cells alters the secretome of cancer cells and results in the inhibition of peritumoral lymphangiogenesis during tumor progression^16^.

In addition, ELK3-targeted mutant mice develop dilated LVs and die after birth due to the accumulation of chyle in the thoracic cage, which results in respiratory failure^17^. These results suggest that ELK3 plays a major role in LECs. Since there is a prometastatic crosstalk between breast cancer cells and LECs^6^, we hypothesized that ELK3 in LECs regulates the expression of communication factors between LEC and tumor cells that promote tumor progression and metastasis. By analyzing the effect of ELK3-suppressed conditioned medium and exosomes from LECs on cancer cells *in vitro* and *in vivo*, we provide direct evidence that ELK3 activates the expression of pro-oncogenic miRNAs and inhibits the expression of antioncogenic miRNAs that are transferred to cancer cells by exosomes. This study shows for the first time that ELK3 expression in LECs contributes to the dissemination of cancer cells during tumor growth by providing oncogenic miRNAs to tumor cells.

## Results

### The ability of LCM to support the migration and invasion of breast cancer cells *in vitro* was associated with the expression of ELK3 in LECs

**Figure 1:**
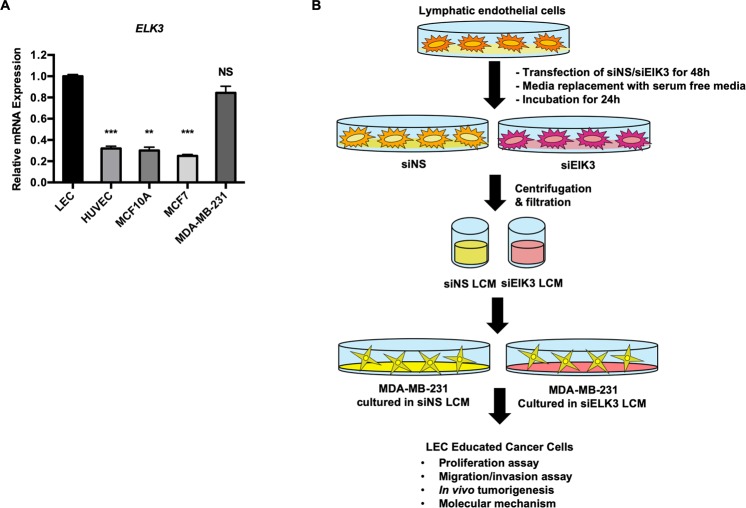
LCM educated tumor model model to study the effect of ELK3 expression levels on the secretome of LECs. (**A**) The relative expression of ELK3 in LECs was compared with that in breast cancer cells by qRT-PCR. (**B**) For the LEC-tumor education model, LCM was prepared from LEC culture. LECs cultured in EBM-2 medium were transfected with nonspecific siRNA (siNS) or an siRNA targeting ELK3 (siELK3) for 24 h, and the cells were further cultured in SFM for 24 h to allow the factors secreted by LECs to accumulate. The medium collected at this time is referred to as siNS LCM or siELK3 LCM, and 30% or 70% LCM coculture medium (LCM: DMEM = 3:7 or 7:3) was applied to MDA-MB-231 cells to educate the cancer cells. Error bars represent the standard error from three independent experiments, and each experiment was performed using triplicate samples.

As an initial step to investigate the role of ELK3 in LECs during tumor development, we first examined the relative expression level of ELK3 in LECs compared to the basal type of breast cancer cells, in which ELK3 is highly expressed and plays an essential role in mediating metastasis^13^. Quantitative analysis revealed that LECs expressed a significant amount of ELK3 that was comparable to that observed in the basal breast cancer cell line MDA-MB-231 (Fig. 1A). To investigate the role of ELK3 expressed in LECs in the ability of LCM to support the oncogenicity of MDA-MB-231 cells *in vitro* and *in vivo*, LCM-educated MDA-MB-231 cells were prepared by the process described in Fig. 1B. LECs cultured in EGM-2 medium were transfected with nonspecific siRNA (siNS) or an siRNA targeting ELK3 (siELK3) for 48 h, and the cells were further cultured in serum free media (SFM) (EBM-2) for 24 h to allow the factors secreted by LECs to accumulate. The medium collected at that time was referred to as siNS LCM or siELK3 LCM. Then, 70% or 30% LCM coculture medium (LCM: DMEM = 7:3 or 3:7) was applied to MDA-MB-231 cells to educate the cancer cells.

**Figure 2:**
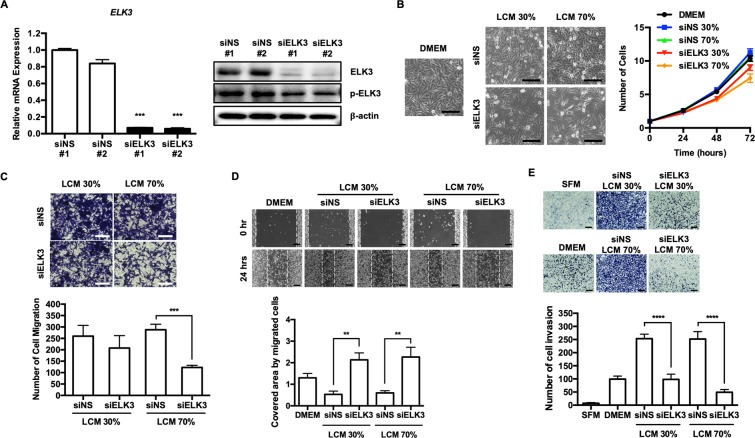
The role of LCM in supporting the migration and invasion of breast cancer cells *in vitro* was associated with the expression level of ELK3 in LECs. (**A**) The suppression of ELK3 in LECs via the transfection of a siRNA targeting ELK3 was confirmed by qRT-PCR and immunoblot analysis. (**B**) MDA-MB-231 cell proliferation was assessed in DMEM, 30% siNS LCM, 30% siELK3, 70% siNS and 70% siELK3. MDA-MB-231 cell proliferation in 30% or 70% siELK3 LCM was significantly lower than that in DMEM, whereas the cell proliferation of MDA-MB-231 cells in 30% or 70% siNS LCM was slightly higher than that observed in DMEM. Scale bar represents 100 μm. (**C**) The effect of siNS or siELK3 LCM on the migration of MDA-MB-231 cells was analyzed by a transwell assay. MDA-MB-231 cells educated in the indicated LCM were cultured in a transwell chamber for 24 h. The migrated cells were stained with crystal violet (Left) and quantified (Right). Scale bar represents 100 μm. (**D**) A scratch wound healing assay was performed to examine the effect of siNS or siELK3 LCM on the migration of MDA-MB-231 cells (Left). The cells were cultured in the indicated LCM, and migration was observed 24 h after scratching (Left) and photographed (Right). Scale bar represents 200 μm. (**E**) Matrigel invasion of MDA-MB-231 cells toward EGF in the presence of the indicated LCM. Scale bar represents 200 μm. Error bars represent the standard error from three independent experiments, and each experiment was performed using triplicate samples. *P < 0.05, **P < 0.01 ***P < 0.001 and ****P < 0.0001 (Student’s t-test).

Since the transfection of siELK3 into LECs successfully suppresses ELK3 expression at both the RNA and protein levels (Fig. 2A), we next examined the effect of siNS LCM or siELK3 LCM on the proliferation of MDA-MB-231 cells. Whereas the proliferation rate of MDA-MB-231 cells cultured in siNS LCM was similar to that of cells cultured in DMEM, the proliferation rate was significantly decreased when the cells were cultured in siELK3 LCM (Fig. 2B). We next analyzed the effect of siNS or siELK3 LCM on the migration of MDA-MB-231 cells by a transwell assay. MDA-MB-231 cells educated in the indicated LCM were cultured in a transwell chamber for 24 h, and the migrated cells were stained with crystal violet. Although the migration of cells cultured in 30% siELK3 LCM was similar to that of cells cultured in 30% siNS LCM, the migration of MDA-MB-231 cells cultured in 70% siELK3 LCM was significantly lower than that of cells cultured in 70% siNS LCM (Fig. 2C). The effect of ELK3 suppression in LEC on LCM activity to promote the migration of MDA-MB-231 cells was further confirmed by a scratch wound healing assay. As shown in Fig. 2D, 30% or 70% siNS LCM increased the migration ability of MDA-MB-231 cells compared to that of cells grown in DMEM. Notably, the migration of MDA-MB-231 cells cultured in 30% or 70% siELK3 LCM was slower than that of cells cultured in DMEM. Besides MDA-MB-231 cells, the migration of other triple negative breast cancer cells such as Hs578T and BT20 was also promoted by LCM and the effect of LCM on the migration was significantly diminished by the suppression of ELK3 in LECs (Fig. S2).

Similar to the migration results, the suppression of ELK3 expression in the LECs significantly diminished the ability of LCM to enhance the invasion of MDA-MB-231 cells *in vitro* (Fig. 2E). The treatment of siELK3 LCM suppressed ERK1/2 or STAT3 signaling pathway of MDA-MB-231, which are representative oncogenic signaling pathways of various cancers (Fig. S3). Based on these results, we concluded that the expression level of ELK3 in LECs is associated with the ability of LCM to support the oncogenicity of MDA-MB-231 cells *in vitro*.

### LCM supports primary tumor progression, and the suppression of ELK3 in LECs can block the pro-oncogenic effect of LCM *in vivo*

**Figure 3:**
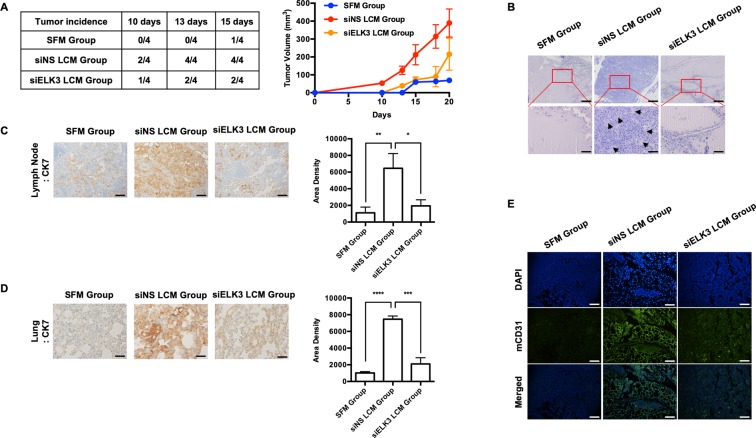
The suppression of ELK3 in LECs diminishes the ability of LECs to promote tumor growth *in vivo*. Athymic nude mice (female, 5–6 weeks of age, 18–20 g) were pretreated with LCM harvested from siNS- or siELK3-transfected LECs or SFM subcutaneously for 2 weeks, as described in the Material and Methods. Then, 2 × 10^6^ MBA-MB-231 cells were mixed with 3.7 × 10^6^ siNS- or siELK3-transfected LECs and Matrigel and inoculated into the mammary fat pads of the corresponding nude mice. MDA-MB-231 cells with siNS LECs were inoculated into siNS LCM-pretreated mice (siNS LCM group), and MDA-MB-231 cells with siELK3 LECs were inoculated into siELK3 LCM-pretreated mice (siELK3 LCM group). For the control, 2 × 10^6^ MDA-MB-231 cells were inoculated into SFM-pretreated mice (SFM group). (**A**) Tumor incidence was examined in four mice from each group on the indicated days after inoculation (Left). Tumor size was measured at the indicated date after inoculation and presented as a graph (Right). (**B**) Matrigel plugs excised from mice 20 days after inoculation were fixed and stained with H&E. Scale bar represents 50 μm. LNs (**C**) or lungs (**D**) were isolated from mice from each group, and immunohistochemical staining was performed to detect human cytokeratin-7 (CK7). Scale bar represents 50 μm. (**E**) LNs from each group were analyzed for the expression of mouse CD31 by immunofluorescence staining. Scale bar represents 50 μm. Error bars represent the standard error from three independent experiments, and each experiment was performed using triplicate samples. *P < 0.05, **P < 0.01 ***P < 0.001 and ****P < 0.0001 (Student’s t-test).

To evaluate whether ELK3 expressed in LECs contributes to the ability of LCM to accelerate the metastasis of MDA-MB-231 cells *in vivo*, we prepared three groups of mice as follows: the “SFM group” included animals pretreated with serum-free medium; the “siNS LCM group” included animals pretreated with LCM harvested from siNS-transfected LECs; and the “siELK3 LCM group” included animals pretreated with LCM harvested from siELK3-transfected LECs. The body weight of mouse was not affected by two weeks of LCM (or SFM) pretreatment (Fig. S4). Then, MDA-MB-231 cells were injected alone into the animals in the “SFM group” as a control, and siNS- or siELK3-transfected LECs were co-injected with MDA-MB-231 cells into animals in the “siNS LCM group” or “siELK3 LCM group”, respectively. We first assessed primary tumor progression every 5 days. As shown in Fig. 3A (left table), the “siELK3 LCM group” developed primary tumors in two of four mice, whereas all four injected mice developed primary tumors in the “siNS LCM group”. Only one of four mice developed a primary tumor in the “SFM group”. In contrast to the “siNS LCM group”, which showed dramatic increases in the tumor growth rate and the final tumor volume, the “siELK3 LCM group” showed a tumor growth rate comparable to that of the “SFM group” until 18 days after xenografting (Fig. 3A right). H&E staining of primary tumor sections further supported the observation that primary tumors in the “siELK3 LCM group” were poorly developed compared to those of the “siNS LCM group”, which suggests that the suppression of ELK3 in LECs restrained the promoting effect of LECs on tumor progression in MDA-MB-231 cells (Fig. 3B). We next assessed metastasis in each group by performing immunohistochemistry with an anti-cytokeratin 7 (CK7) antibody on sections from the axillary LN and lung. Consistent with the results obtained for primary tumor progression, the population of CK7-positive cells in the LN and lung samples harvested from the “siELK3 LCM group” was much smaller than that from the “siNS LCM group” (Fig. 3C,D). CD31 endothelial cell marker staining in the LN further revealed that the large population of CD31-positive cells in the LN of the “siNS LCM group” was significantly decreased in the “siELK3 LCM group”, comparable to that of the “SFM group” (Fig. 3E). Based on these results, we concluded that ELK3 is an essential regulator of oncogenic activity in the secretome of LECs.

### ELK3 determines the pro-oncogenic activity of exosomes secreted from LECs

**Figure 4:**
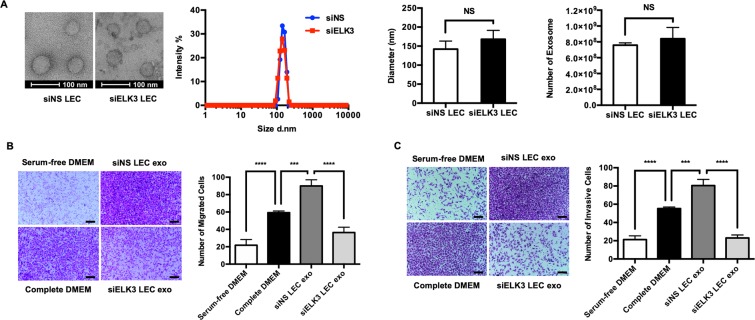
Suppression of ELK3 in LECs diminishes the ability of LEC-derived exosomes to promote cancer cell migration and invasion *in vitro*. (**A**) Exosomes purified from siNS- or siELK3-transfected LECs were examined via electron microscopy (Left). Scale bar represents 100 nm. The size distribution of the exosomes was measured by DLS (Middle). The total number of exosomes derived from siNS- or siELK3-transfected LECs was analyzed by detecting the expression level of exosomal marker, CD69 (Right). The effect of exosomes purified from siNS- or siELK3-transfected LECs on the migration (**B**) or invasion (**C**) of MDA-MB-231 cells was measured by a transwell assay. Serum-free DMEM and complete DMEM were added to MBA-MB-231 cells as (−) and (+) controls for the migration and invasion assay. Scale bar represents 100 μm. Error bars represent the standard error from three independent experiments, and each experiment was performed using triplicate samples. *P < 0.05, **P < 0.01 ***P < 0.001 and ****P < 0.0001 (Student’s t-test).

Several studies have demonstrated that exosomes derived from tumor or endothelial cells are able to modulate the tumor microenvironment by mediating cell-to-cell communication^18–20^. Therefore, we hypothesized that the pro-oncogenic activity of LCM might be elicited by exosomes released from LECs and that ELK3 plays an essential role in determining the quality of exosomes from LECs. We thus isolated exosomes from siNS- or siELK3-transfected LECs and examined the effect of ELK3 expression on the morphology and size of exosomes. Purified exosomes were visualized by transmission electron microscopy, and their size distribution was evaluated using dynamic light scattering. As shown in Fig. 4A, the suppression of ELK3 did not affect the morphology, size distribution or total number of exosomes released from LECs. To investigate the effect of ELK3 expression in LECs on the migration and invasion of cancer cells, a transwell assay was performed with MDA-MB-231 cells in the presence of exosomes isolated from siNS or siELK3 LECs. The siNS-LEC exosome-treated groups had enhanced migration and invasion abilities compared to those of the siELK3-LEC exosome-treated groups (Fig. 4B,C), suggesting that the contents of siNS-LEC exosomes stimulate MDA-MB-231 cells to adopt a more metastatic status and that the suppression of ELK3 in LECs prevented the contents from being released by exosomes.

### ELK3 determines the miRNA contents of exosomes derived from LECs

**Figure 5:**
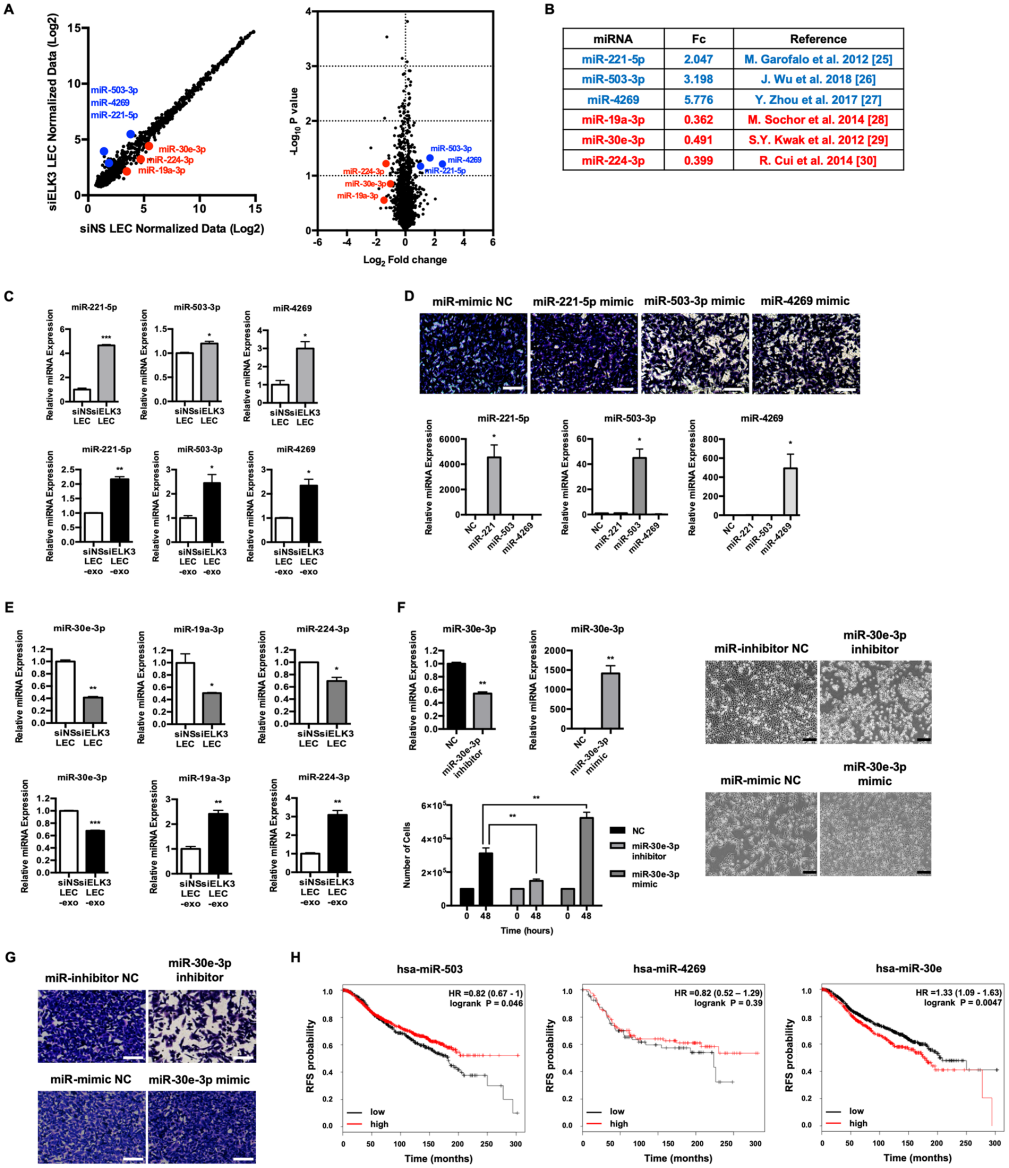
ELK3 determines the miRNA contents of exosomes derived from LECs. (**A**) miRNA expression comparison between siNS LECs and siELK3 LECs. (Left) Scatter plot of the miRNA microarray analysis. The axes of scatter plot are normalized data from the samples. (Right) Volcano plot representing the p-values (Y axis) for the observed differences in miRNA expression (X axis). Three representative upregulated miRNAs were labeled in blue, and 3 representative downregulated miRNAs were labeled in red. (**B**) The analyzed representative miRNAs, fold changes from the miRNA microarray results and miRNA status as a tumor suppressor miRNA or onco-miRNA. (**C**) The intracellular and exosomal levels of 3 miRNAs that were selected as candidate antioncogenic miRNAs were analyzed by qRT-PCR. (**D**) Transwell assay of MDA-MB-231 cells that were transfected with the indicated miRNA mimics (Upper). The expression level of each miRNA was analyzed by qRT-PCR (Lower). Scale bar represents 100 μm. (**E**) The intracellular and exosomal levels of 3 miRNAs selected as candidate pro-oncogenic miRNAs were analyzed by qRT-PCR. (**F**) The effect of miR-30e-3p mimic or miR-30e-3p inhibitor on the proliferation of MDA-MB-231. The expression level of miR-30e-3p for each treatment was analyzed by qRT-PCR and the effect of each miRNA on the cell proliferation was assessed by cell counting at the indicated time (Left). Cell morphology was visualized via light microscopy at 48 h after transfection of each miRNAs (Right). Scale bar represents 200 μm. (**G**) Transwell assay of MDA-MB-231 cells that were transfected with the miR-30e-3p mimic or miR-30e-3p inhibitor. Scale bar represents 100 μm. (**H**) Kaplan-Meier plots for miR-503, miR-4269 and miR-30e. The plots for miR-503 and miR-30e were analyzed with all subtypes of breast cancer patients from the METABRIC dataset, and the plot for miR-4269 was analyzed with triple-negative breast cancer patients from the METABRIC dataset. Error bars represent the standard error from three independent experiments, and each experiment was performed using triplicate samples. *P < 0.05, **P < 0.01 ***P < 0.001 and ****P < 0.0001 (Student’s t-test).

It is known that one of the main constituents of exosomes is miRNA, and most miRNAs are expressed at similar levels in cells and exosomes^21,22^. To identify miRNAs from LECs that are regulated by ELK3 and can affect tumor progression, we compared the miRNA profiles of siELK3-transfected LECs with those of siNS-transfected LECs. Based on the data from the 6,658 miRNA probe sets, 89 probe sets were upregulated in siELK3 LECs compared to siNS LECs, and 78 probe sets were downregulated based on the normalized fold change (FC > 1.5 or <0.67) (Fig. S5). A scatter plot and volcano plot were generated to visualize the differential expression between siNS LECs and siELK3 LECs (Fig. 5A). Based on gene expression fold changes in the miRNA array and published data, we selected 6 miRNAs for further analysis (Fig. 5B). We first confirmed that the levels of the selected miRNAs in exosomes were consistent with the results of the miRNA array. As shown in 5C, the 3 miRNAs (miR-221-5p, miR-503-3p, and miR-4269) that were selected as tumor-suppressive miRNAs^23–25^ were detected at high levels in exosomes derived from siELK3 LECs compared to siNS LECs (Fig. 5C). Since an increasing number of studies have demonstrated that the pro- or anti-oncogenic activity of miRNAs depends largely on the cellular context and the target genes, we investigated the effects of these 3 miRNAs on the migration ability of MDA-MB-231 cells. When the miRNA mimic of each miRNA was delivered to MDA-MB-231 cells by Lipofectamine, the migration ability of MDA-MB-231 cells was decreased by the transfection of the miR-503-3p and miR4269 mimics (Fig. 5D). These results indicate that ELK3 from LECs inhibits the expression of tumor-suppressive miRNAs, and the suppression of ELK3 expression results in increases in these miRNAs, which can be delivered by exosomes from LECs to tumors. We next analyzed the expression of these 3 miRNAs (miR-19a, miR-30e-3p, and miR-224-3p) that were selected as onco-miRs^26–28^. Although the expression patterns of miR-19a-3p and miR-224-3p in exosomes were not consistent with the intracellular levels, we noted that miR-30e-3p was decreased both at the intracellular level and in exosomes from siELK3 LECs (Fig. 5E). We confirmed that miR-30e-3p functions as an onco-miR in MDA-MB-231 cells by examining the effect of the miR-30e-3p mimic to promote the cell proliferation of MDA-MB-231 cells (Fig. 5F). The ability of the miR-30e-3p inhibitor to suppress migration and the ability of miR-30e-3p mimic to promote migration further supports the conclusion that miR-30e-3p functions as an onco-miR in MDA-MB-231 cells (Fig. 5G). These results suggest that ELK3 expression is positively correlated with the expression of oncogenic miRNAs, such as miR-30e-3p, in LECs. Finally, we examined the relation between patient survival and miRNA expression using Kaplan-Meier analysis. As shown in Fig. 5H, breast cancer patients with high miR-503 expression survive longer than patients with low miR-503 expression. Similarly, miR-4269 shows results similar to those obtained with miR-503, with a higher p-value. These results suggest the possibility that miR-503 and miR-4269 derived from LECs function as tumor suppressors in breast cancer. On the other hand, analysis of miR-30e shows that a high level of miR-30e exerts adverse effects on the survival of breast cancer patients, with a low p-value. Since miR-30e is highly expressed in control LECs and suppressed in siELK3 LECs, it is plausible to hypothesize that the decreased miR-30e concentration in exosomes from siELK3 LECs is one mechanism for the loss of pro-oncogenic activity in LCM from siELK3 LECs.

## Discussion

Under pathological conditions, such as neoplasia, the dramatic expansion of peripheral LVs occurs through the enlargement of existing vessels and the induction of de novo lymphangiogenesis^29^. An increased lymphatic vessel density in and around the tumor is predicted to facilitate metastatic dissemination and the spreading of tumor cells and consequently correlates with poor outcomes in many solid tumors^30^. Although LECs within the tumor microenvironment are predicted to promote tumor growth by communicating with tumor cells, the mechanisms behind the prometastatic activity of tumor-associated LECs are poorly understood. Recently, it was reported that the education of LECs with tumor-conditioned medium enhances the secretion of lymphangiocrine factors, including EGF and PDGF-BB, that promote tumor cell proliferation, angiogenesis and pericyte infiltration^3^.

In this study, we demonstrate that LECs produce exosomes that are able to promote breast tumor progression and that ELK3 expressed in LECs promotes the expression of pro-oncogenic miRNAs and suppresses antioncogenic miRNAs, which are transferred to tumor cells via exosomes.

ELK3 is a strong transcriptional repressor due to the activity of two repressor domains, and it is converted to a transcriptional activator via phosphorylation by the RAS-mitogen activated kinase (RAS-MAPK) signaling pathway^31^. This feature of ELK3 is a hurdle for investigating the underlying mechanism of ELK3, especially for identifying the direct downstream targets. We predict that ELK3 functions as a transcriptional activator in LECs to regulate miRNA expression because the phosphorylated form of ELK3 was detected in the cell extract from LECs (Fig. 2A). However, an antibody to detect the non-phosphorylated form of ELK3 is not commercially available yet. For this reason, we cannot rule out the possibility that only a portion of the ELK3 is phosphorylated to function as a transcriptional activator, and residual non-phosphorylated ELK3 functions as a transcriptional repressor.

We did not clarify whether miRNAs secreted from LECs are direct targets of ELK3. Since the suppression of ELK3 increases the expression of antioncogenic miRNAs, such as miR-503-3p and miR-4269, and decreases the expression of pro-oncogenic miRNAs, such as miR-30e-3p, it is plausible to hypothesize that the transcriptional activator form and the repressor form of ELK3 exist simultaneously in LECs. This hypothesis will be verified through in-depth molecular biological experiments, including chromatin immunoprecipitation and luciferase assays.

In addition to MDA-MB-231, siNS or siELK3 LCMs have similar effects on other triple negative breast cancer cells such as Hs578T and BT20 (Fig. S2). However, it is not conclusive whether the identical miRNAs secreted from LECs, which have pro-oncogenic or anti-oncogenic activity on MDA-MB-231, have similar effect on Hs578T or BT20. Indeed, when we analyzed the oncogenic activity of miRNA mimic of miR-221-5p, miR-503-3p and miR-4269 on Hs578T and BT20, miR-4269 mimic did not have any effect on either cell, whereas miR-503-3p and miR-221-5p mimic showed anti-oncogenic activity on Hs578T and BT20, respectively (data not shown).

The expression level of ELK3 in MDA-MB-231 cells is associated with peritumoral lymphangiogenesis^16^. The knockdown of ELK3 expression in MDA-MB-231 cells results in the suppression of peritumoral lymphatic vessel development, possibly through low vascular endothelial growth factor - C (VEGF-C) expression. Since ELK3 has pro-oncogenic activity for both cancer cells and LECs, ELK3 might modulate the tumor microenvironment by fine-tuning communication between LECs and tumor cells. In this context, ELK3 is a promising candidate for developing an anticancer drug that prevents metastasis by controlling the tumor microenvironment.

Compared to tumor cells, which exhibit heterogeneity due to genetic mutations and epigenetic alterations, cells that reside in the tumor microenvironment are genetically and epigenetically more stable^32^. Therefore, targeting noncancer cells, such as LECs, can serve as an effective strategy to defeat cancer.

In summary, in this study, we experimentally established that (i) exosomes derived from LECs have pro-oncogenic activity; (ii) ELK3 determines the constituents of exosomes derived from LECs; and (iii) ELK3 from LECs regulates the expression of microRNAs that can promote or repress tumor progression and metastasis.

## Material and Methods

### Cell culture

LECs were purchased from Lonza Biologics (Basel, Switzerland) and grown in EGM-2 medium with supplements (Lonza Clonetics, Basel, Switzerland) in the presence of 5% CO_2_ at 37 °C. MDA-MB-231, Hs578T, BT20 breast cancer cells were purchased from ATCC (Manassas, VA, USA) and cultured in Dulbecco’s modified Eagle’s medium (DMEM) supplemented with 10% fetal bovine serum (FBS) (Gibco BRL, Waltham, MA, USA) and 1% penicillin/streptomycin (Invitrogen, Carlsbad, CA, USA).

### Knockdown of ELK3 and preparation of LEC-conditioned medium

Nonspecific control siRNA (siNS, D-001810-10) and human ELK3 siRNA (siELK3, L-010320-00-0005) were obtained from Dharmacon, Inc. (Chicago, IL, USA). siNS or siELK3 was transfected into LECs using Lipofectamine 2000 (Invitrogen, CA, USA) according to the manufacturer’s instructions. To prepare LEC-conditioned medium (LCM), the complete culture medium from LECs was replaced with serum-free medium (SFM, EBM-2) 24 h after siRNA transfection. The culture medium was collected 24 h after medium replacement and filtered through a 0.2 μm syringe filter (Corning, Tewksbury, MA, USA). LCM was stored in aliquots at −80 °C.

### Western blot analysis

For protein analysis, the cells were washed twice with cold phosphate-buffered saline (PBS, Gibco BRL, Waltham, MA, USA) and lysed in cell lysis buffer (Cell Signaling Technology, Danvers, MA, USA). Total cell extracts were resolved by sodium dodecyl sulfate-polyacrylamide gel electrophoresis, transferred to polyvinylidene fluoride (PVDF) membranes (Bio-Rad, Hercules, CA, USA), and blotted with antibodies against β-actin (Santa Cruz Biotechnology, Santa Cruz, CA, USA), ELK3 (Novus, Littleton, CO, USA), and phospho-ELK3 (GeneTex, Irvine, CA, USA). Immunoreactivity was detected with enhanced chemiluminescence (Thermo Fisher Scientific, Rochester, NY, USA).

### Cell proliferation assays

An MDA-MB-231 cell proliferation assay was performed by counting cell numbers. First, MDA-MB-231 cells were seeded into 6-well plates at a density of 5 × 10^4^ cells per well and allowed to adhere overnight. Next, the medium was replaced, and cell numbers were counted at 24 h, 48 h and 72 h after medium replacement. All samples were assayed in triplicate, and the mean for each experiment was statistically analyzed.

### Cell migration assay and invasion assay

For the migration assay, MDA-MB-231 cells were seeded into 6-well plates at a density 2 × 10^5^ cells per well. The following day, a uniform scratch was made down the center of the well using a 100 μl micropipette tip. Cell migration was quantified by measuring the ratio of the migration area to the total area of the wound gap. For transwell-based migration assays, MDA-MB-231, Hs578T and BT20 cells were seeded in the upper chamber well of a transwell plate (Corning, Tewksbury, MA, USA), and LCM was placed in the lower portion of the chamber well. After incubation for 24 h, the cells that migrated to the bottom chamber were removed from the underside of the membrane, fixed in 4% paraformaldehyde (Santa Cruz Biotechnology, Santa Cruz, CA, USA), and stained with crystal violet. For the invasion assays, 8 × 10^4^ cells were placed onto a transwell membrane coated with 50 μl of Matrigel (BD Biosciences, San Jose, CA, USA) and then incubated for 36 h. The migrated cells were processed as described above.

### LCM-induced metastasis model

All animal experiments were approved by the Institutional Animal Care and Use Committee of laboratory animal research center in CHA university (IACUC-160014). We confirm that all experiments were performed in accordance with relevant guidelines and regulations. We pretreated athymic nude mice (female, 5–6 weeks of age, 18–20 g) by injection with 50 μl of LCM harvested from siNS- or siELK3-transfected LECs or SFM subcutaneously for 2 weeks, as described previously^33^. Then, 1 × 10^6^ of MDA-MB-231 cells and 2 × 10^6^ of siNS- or siELK3-transfected LECs were mixed with 50 μl of Matrigel (high concentration, BD Biosciences, San Jose, CA, USA), and the mixture was orthotopically inoculated into the mammary fat pads of the mice. The tumor size was measured by using a caliper, and the volume was calculated by the following formula: V = 0.52 × (tumor length) × (tumor width)^2^. After 4 weeks, all mice were sacrificed and the primary tumor, the axillary lymph node (LN) and the lung were harvested.

### Histology

We performed 3,3′ diaminobenzidine (DAB)-mediated immunohistochemistry with human cytokeratin 7 (hCK7) antibodies (Dako, Glostrup, DK) on sections of paraffin-embedded lung and LN tissue. For immunofluorescence staining of mouse CD31 (BD Biosciences, San Jose, CA, USA), sections of paraffin-embedded tissues were blocked with 5% normal goat serum in 1 × PBS with 0.3% Triton (PBST) for 1 h at room temperature and then treated with primary antibodies overnight at 4 °C. After 3 rinses with PBST, the tissues were incubated with an Alexa Fluor 488 goat anti-rabbit antibody (Cell Signaling Technology, Danvers, MA, USA) for 1 h at room temperature, and immunofluorescence was examined using a Zeiss AxioImager M1 fluorescence microscope (Carl Zeiss, Oberkochen, Germany).

### Exosome purification

The culture medium was collected and centrifuged at 3000 g for 15 min. The supernatant was filtered through a 0.22 μm filter (Millipore, Burlington, MA, USA). The appropriate volume of total exosome isolation reagent (Invitrogen, Carlsbad, CA, USA) was added to the filtered culture medium and mixed well by inverting. After refrigeration overnight, the mixture was centrifuged at 10000 g for 1 h, and all supernatant was removed by aspiration. Exosome pellets were resuspended with cold PBS. Total number of exosomes was determined using the ExoELISA Exosome Quantification Assay Kit (System Biosciences, Palo Alto, CA, USA), which detects general exosome marker, CD63, based on the ELISA method.

### miRNA microarray and miRNA expression analysis

Total RNA was extracted using the TRIzol Reagent (Invitrogen, Carlsbad, CA, USA) according to the manufacturer’s instructions. RNA quality and quantity were assessed by Agilent Bioanalyzer 2100 analysis. Starting with 250 ng of total RNA, the labeling process began with poly-A tailing of each RNA strand using poly-A polymerase, followed by ligation of a biotin-labeled 3DNA dendrimer. The biotinylated RNA strands were hybridized at 48 °C for 18 h on an Affymetrix GeneChip miRNA 4.0 Array (Affymetrix, Santa Clara, CA, USA). The GeneChip miRNA 4.0 Array was washed and stained in the Affymetrix Fluidics Station 450. Fluorescence signals amplified by the branched structure of the 3DNA dendrimer were scanned using the Affymetrix GeneChip Scanner 3000 7G. The arrays were analyzed using an Agilent scanner with the associated software. miRNA expression levels were calculated with Expression Console 1.4 (Affymetrix, Santa Clara, CA, USA). The relative signal intensities of each miRNA were generated using the Robust Multi-Array Average algorithm. Target prediction was analyzed using the miRBase, miRDB, TargetScan and microRNA.org databases. To quantify miRNA expression levels, total RNA was converted to cDNA, and real-time reverse transcriptase-polymerase chain reaction (RT-PCR) was performed following the protocol from the HB miR Multi Assay Kit (Heim Biotek, Gyeonggi-do, Korea). Mimic and inhibitor miRNAs were purchased from Genolution, Inc. (Seoul, Korea).

### Statistical analysis

Samples were analyzed with unpaired Student’s t-test assuming equal variances (two-tailed) between the two independent groups. All statistical analyses were performed using GraphPad Prism 6 (GraphPad Prism, San Diego, CA, USA). P-values less than 0.05 were considered statistically significant.

## Supplementary information

Supplementary information

## References

[1] Alitalo A, Detmar M (2012). Interaction of tumor cells and lymphatic vessels in cancer progression. Oncogene.

[2] Piltonen M (2011). Vascular endothelial growth factor C acts as a neurotrophic factor for dopamine neurons *in vitro* and *in vivo*. Neuroscience.

[3] Lee E, Pandey NB, Popel AS (2014). Pre-treatment of mice with tumor-conditioned media accelerates metastasis to lymph nodes and lungs: a new spontaneous breast cancer metastasis model. Clin Exp Metastasis.

[4] Swartz MA, Lund AW (2012). Lymphatic and interstitial flow in the tumour microenvironment: linking mechanobiology with immunity. Nature reviews. Cancer.

[5] Yun JH (2014). Incident light adjustable solar cell by periodic nanolens architecture. Scientific reports.

[6] Lee E, Pandey NB, Popel AS (2014). Lymphatic endothelial cells support tumor growth in breast cancer. Scientific reports.

[7] Chen ST (2008). Breast tumor microenvironment: proteomics highlights the treatments targeting secretome. J Proteome Res.

[8] Alitalo K (2011). The lymphatic vasculature in disease. Nat Med.

[9] Jiang X (2017). Exosomal microRNA remodels the tumor microenvironment. PeerJ.

[10] Mathivanan S, Ji H, Simpson RJ (2010). Exosomes: extracellular organelles important in intercellular communication. Journal of proteomics.

[11] Zhang L (2015). Microenvironment-induced PTEN loss by exosomal microRNA primes brain metastasis outgrowth. Nature.

[12] Maira SM, Wurtz JM, Wasylyk B (1996). Net (ERP/SAP2) one of the Ras-inducible TCFs, has a novel inhibitory domain with resemblance to the helix-loop-helix motif. The EMBO journal.

[13] Kong SY (2016). The ELK3-GATA3 axis orchestrates invasion and metastasis of breast cancer cells *in vitro* and *in vivo*. Oncotarget.

[14] Lee JH (2016). STAT3-induced WDR1 overexpression promotes breast cancer cell migration. Cellular signalling.

[15] Yang H (2015). ETS family transcriptional regulators drive chromatin dynamics and malignancy in squamous cell carcinomas. eLife.

[16] Oh N (2017). The role of ELK3 to regulate peritumoral lymphangiogenesis and VEGF-C production in triple negative breast cancer cells. Biochemical and biophysical research communications.

[17] Ayadi A (2001). Net-targeted mutant mice develop a vascular phenotype and up-regulate egr-1. The EMBO journal.

[18] Bovy N (2015). Endothelial exosomes contribute to the antitumor response during breast cancer neoadjuvant chemotherapy via microRNA transfer. Oncotarget.

[19] Fu H, Yang H, Zhang X, Xu W (2016). The emerging roles of exosomes in tumor-stroma interaction. Journal of cancer research and clinical oncology.

[20] Kahlert C, Kalluri R (2013). Exosomes in tumor microenvironment influence cancer progression and metastasis. Journal of molecular medicine.

[21] Hessvik NP, Phuyal S, Brech A, Sandvig K, Llorente A (2012). Profiling of microRNAs in exosomes released from PC-3 prostate cancer cells. Biochimica et biophysica acta.

[22] Mittelbrunn M (2011). Unidirectional transfer of microRNA-loaded exosomes from T cells to antigen-presenting cells. Nature communications.

[23] Garofalo M, Quintavalle C, Romano G, Croce CM, Condorelli G (2012). miR221/222 in cancer: their role in tumor progression and response to therapy. Current molecular medicine.

[24] Wu J (2018). miR-503 suppresses the proliferation and metastasis of esophageal squamous cell carcinoma by triggering autophagy via PKA/mTOR signaling. International journal of oncology.

[25] Zhou Y (2017). TEAD1/4 exerts oncogenic role and is negatively regulated by miR-4269 in gastric tumorigenesis. Oncogene.

[26] Sochor M (2014). Oncogenic microRNAs: miR-155, miR-19a, miR-181b, and miR-24 enable monitoring of early breast cancer in serum. BMC cancer.

[27] Kwak SY (2015). Ionizing radiation-inducible miR-30e promotes glioma cell invasion through EGFR stabilization by directly targeting CBL-B. The FEBS journal.

[28] Cui R (2015). MicroRNA-224 promotes tumor progression in nonsmall cell lung cancer. Proceedings of the National Academy of Sciences of the United States of America.

[29] Stacker SA (2014). Lymphangiogenesis and lymphatic vessel remodelling in cancer. Nature reviews. Cancer.

[30] Pasquali S (2013). Lymphatic biomarkers in primary melanomas as predictors of regional lymph node metastasis and patient outcomes. Pigment cell & melanoma research.

[31] Giovane A, Pintzas A, Maira SM, Sobieszczuk P, Wasylyk B (1994). Net, a new ets transcription factor that is activated by Ras. Genes & development.

[32] Koren S, Bentires-Alj M (2015). Breast Tumor Heterogeneity: Source of Fitness, Hurdle for Therapy. Molecular cell.

[33] Lee E, Koskimaki JE, Pandey NB, Popel AS (2013). Inhibition of lymphangiogenesis and angiogenesis in breast tumor xenografts and lymph nodes by a peptide derived from transmembrane protein 45A. Neoplasia.

