# Supplementary material for: ELK3 expressed in lymphatic endothelial cells promotes breast cancer progression and metastasis through exosomal miRNAs
Source: Sci Rep. 2019 Jun 10;9:8418. doi: 10.1038/s41598-019-44828-6 (PMC6557839; doi:10.1038/s41598-019-44828-6)
Supplement: Supplementary file 1 — Supplementary information [file 41598_2019_44828_MOESM1_ESM.pdf]

Supplementary information

ELK3 expressed in lymphatic endothelial cells promotes breast cancer progression and metastasis through exosomal miRNAs

Kwang-Soo Kim[1], Ji-In Park, Nuri Oh[1], Hyeon-Ju Cho[1], Ji-Hoon Park[1], Kyung-Soon Park[1*]

[1]Department of Biomedical Science, College of Life Science, CHA University, Seongnam-si, Republic of Korea

[*]Address correspondence to Kyung-Soon Park, PhD, Department of Biomedical Science, College of Life Science, CHA University, 335, Pangyo-ro, Bundang-gu, Seongnam-si, Gyeonggi-do, Republic of Korea, 13488. Tel.: 82-31-881-7144; Fax: 82-31-881-7249; E-mail: kspark@cha.ac.kr

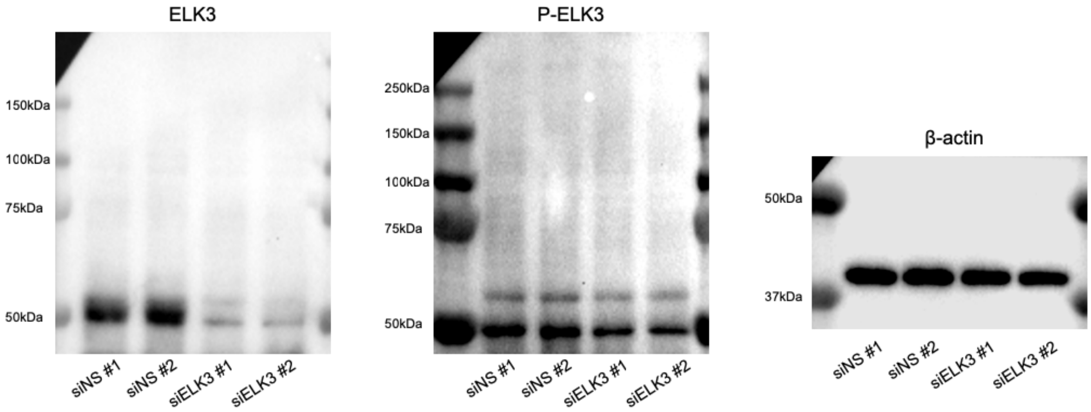

Figure S1. Knockdown of ELK3 expression. Western blot analysis of phospho-ELK3, ELK3 and β-actin in MDA-MB-231 cells expressing non-specific control siRNA (siNS) or ELK3 siRNA (siELK3).

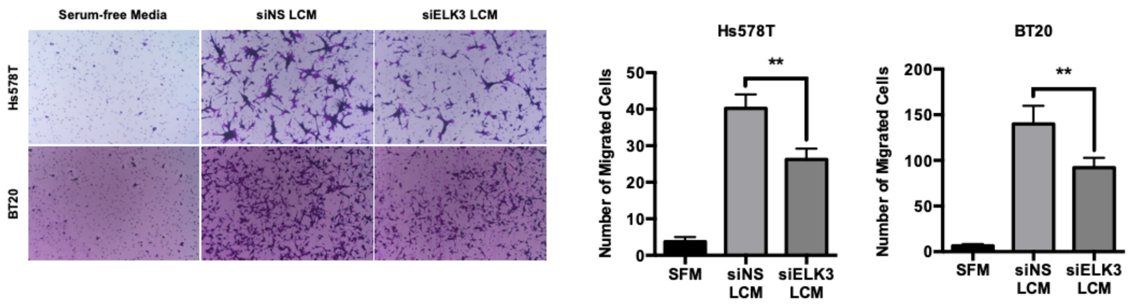

Figure S2. The effect of siNS or siELK3 LCM on the migration of Hs578T or BT20 cells was analyzed by a transwell assay. Hs578T or BT20 cells educated in the indicated LCM were cultured in a transwell chamber for 24 h. The migrated cells were stained with crystal violet (Left) and quantified (Right).

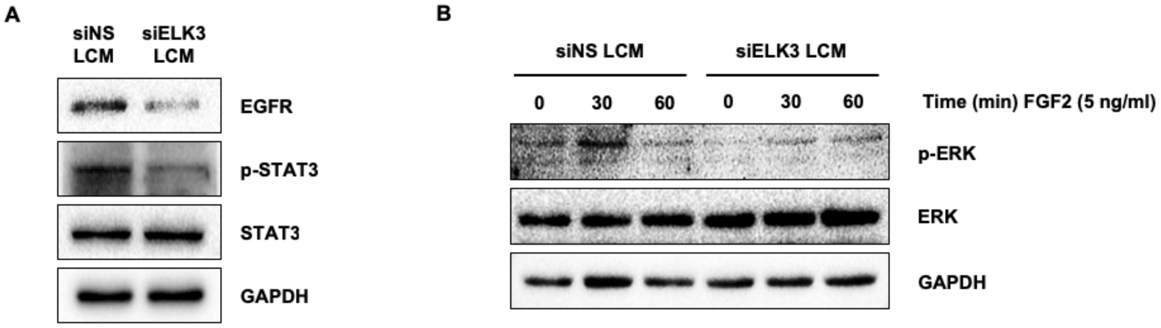

Figure S3. The effect of ELK3 suppressed LCM on the ERK1/2 or stat3 signaling pathway of MDA-MB-231.

(A) The expression of EGFR and phospho-Stat3 of MDA-MB231 that was cultured in siNS or siELK3 LCM for 24 h on was analyzed by immunoblot analysis. (B) The activation of ERK signaling pathway was analyzed by immunoblot analysis of MDA-MB-231 cells that were cultured in the siNS or siELK3 for 24 h and then stimulated with FGF2 (5 ng/ml) for indicated times.

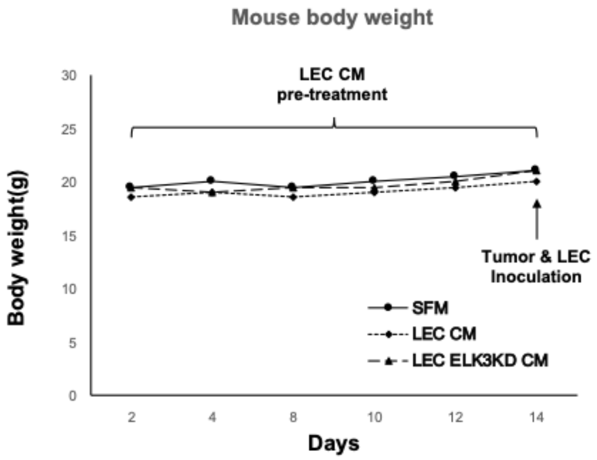

Figure S4. Body weight of mice that were pretreated with LCM harvested from siNS- or siELK3-transfected LECs or SFM subcutaneously for 2 weeks, as described in the Material and Methods.

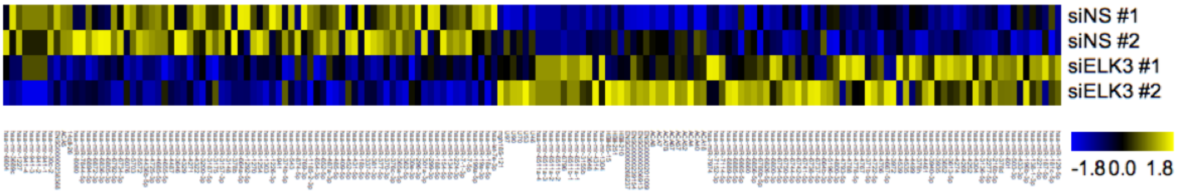

Figure S5. miRNA microarray analysis of siNS or siELK3 transfected LECs. A heatmap was generated from standardized Z-score with expression of each gene. Among the differentially expressed miRNAs, 167 miRNAs (normalized fold change > 1.5 or < 0.67) were represented. Yellow indicates upregulation; blue, downregulation.
